# Trophic Actions of Bone Marrow-Derived Mesenchymal Stromal Cells for Muscle Repair/Regeneration

**DOI:** 10.3390/cells1040832

**Published:** 2012-10-17

**Authors:** Chiara Sassoli, Sandra Zecchi-Orlandini, Lucia Formigli

**Affiliations:** Department of Anatomy, Histology and Forensic Medicine, University of Florence, Largo Brambilla 3, Florence 50134, Italy; Email: csassoli@unifi.it (C.S.); zecchi@unifi.it (S.Z.-O.)

**Keywords:** cell-based therapy, cell proliferation, mesenchymal stromal cells (MSCs), muscle repair/regeneration, myogenic progenitors, paracrine factors

## Abstract

Bone marrow-derived mesenchymal stromal cells (BM-MSCs) represent the leading candidate cell in tissue engineering and regenerative medicine. These cells can be easily isolated, expanded *in vitro* and are capable of providing significant functional benefits after implantation in the damaged muscle tissues. Despite their plasticity, the participation of BM-MSCs to new muscle fiber formation is controversial; in fact, emerging evidence indicates that their therapeutic effects occur without signs of long-term tissue engraftment and involve the paracrine secretion of cytokines and growth factors with multiple effects on the injured tissue, including modulation of inflammation and immune reaction, positive extracellular matrix (ECM) remodeling, angiogenesis and protection from apoptosis. Recently, a new role for BM-MSCs in the stimulation of muscle progenitor cells proliferation has been demonstrated, suggesting the potential ability of these cells to influence the fate of local stem cells and augment the endogenous mechanisms of repair/regeneration in the damaged tissues.

## 1. Introduction

Skeletal and cardiac muscles have received much attention in the last years with respect to their regenerative capacities. It is well known that adult skeletal muscle can efficiently undergo repair/regeneration in response to trauma or degenerative diseases due to the activity of a resident population of muscle progenitors, namely satellite cells [1]. These mononucleated cells are localized underneath the basal lamina of each muscle fiber closely juxtaposed to the sarcolemma, within special niches. Here, these cells lie in a dormant state and start to proliferate and differentiate in response to signals emanating from the damaged fibers and infiltrating inflammatory cells in order to form new myofibers within a few days [2,3,4,5,6]. Unfortunately, satellite cells are relatively scarce within the skeletal muscle tissue, representing about 1%–5% of the total muscle nuclei and, in the case of severe muscle damage, they might not be recruited in a sufficient amount at the site of tissue damage. Moreover, their capacity to accomplish the myogenic program is highly compromised by the induction of the inflammatory response and the excessive fibroblast proliferation and collagen fiber deposition [7,8,9]. Recently, a small population of resident stem/myogenic progenitors (cardiac stem cells, CSCs) have also been identified in the adult heart of mammals including humans, questioning the traditional paradigm of the myocardium as a post-mitotic terminally differentiated tissue [10,11,12,13,14]. These cells are mainly localized into sub-epicardial clusters in the atria, in the ventricular base through the midregion and in the apex [10,11] However, differently from satellite cells, resident CSCs are mainly involved in the maintenance of cardiac tissue homeostasis [15,16,17], showing a limited regenerative potential [18,19,20,21]. Many researchers have thus been devoted to the development of therapeutic strategies for the treatment of degenerative muscle diseases; among them, the stem cell-based therapy is emerging as one of the most promising tools in the field of regenerative medicine [6,20,22,23,24,25,26,27]. The most obvious candidate cells to be transplanted for skeletal muscle regeneration are the satellite cells, whose therapeutic potency has been demonstrated in experimental models of human dystrophy and skeletal muscle injury [22,28,29]. However, their use for regenerative purposes is hindered by several criticisms including: (i) the high heterogeneity of this cell population [30]; (ii) the loss of their myogenic potential upon isolation and *in vitro* expansion [29]; (iii) the predetermination dependent from the source (slow *versus* fast, head *versus* limb muscles) of skeletal muscle fibers [31,32]; (iv) the scarce cell survival in the host tissue [33]; and, (v) the inability to cross the blood vessel wall, restricting their use to the local injection [23]. The therapeutic application of CSCs in the injured myocardium has also some limitations and concerns substantially related to the absence of a full understanding of the biological and immunophenotypical features of these cells and to the difficulties of their culture expansion and implantation [12,34,35,36,37,38,39]. These hurdles have shifted the attention of many researchers in the field of cell-based therapy to other stem cell types, in particular to adult bone marrow-derived mesenchymal stromal cells (BM-MSCs) for the treatment of the damaged muscle. These cells, in fact, possess unique biological properties which render them promising candidate cells to be used in preclinical and clinical settings for tissue repair/regeneration. 

This concise review will focus on the therapeutic applications of BM-MSCs for skeletal and cardiac muscle repair /regeneration, paying particular attention to the mechanisms through which these cells exert their beneficial effects.

## 2. Bone Marrow-Derived Mesenchymal Stromal Cells (BM-MSCs)

### 2.1. Biological Properties

MSCs constitute a rare population of adult stem cells, found *in situ* within all adult mammalian supportive stromal tissue compartments; however, their main source remains the bone marrow where they were first identified over 40 years ago [40,41]. These cells are defined on the basis of their plastic adherence in standard culture condition, a spindle-shaped appearance, their phenotypic characteristics and a capability to be induced to differentiate into adipocytes, osteoblasts and chondrocytes. The phenotype definition requires the expression of CD73 (an ecto-5'-nucleotidae, involved in bone marrow stromal interactions, MSC migration and modulation of adaptive immunity), CD90 (Thy1 antigen, with unknown function) and CD105 (or endoglin, the transforming growth factor (TGF)-β receptor III implicated in MSC chondrogenic differentiation) together with the lack of expression of CD11b and CD14 (monocyte and macrophage markers), CD34 (hematopoietic progenitor and endothelial cell marker), CD45 (leukocyte marker) CD19 or CD79a (B cell marker) and human leukocyte antigen (HLA)-DR surface molecules [42,43]. Despite these well-established criteria for defining MSCs, their isolation is hindered by the possible contamination of non-mesenchymal cells, resulting in a heterogeneous cell population with unpredictable MSC content [44]. Therefore, alternative preparation strategies have been recently postulated to improve the purity of the cell culture, such as the use of novel antibodies with specific reactivity against cell surface molecules highly expressed by MSCs (Stro-1, Stro-3, Stro-4, CD71, VCAM-1) [44,45]. MSCs possess many biological properties that make these cells ideal candidates for tissue engineering and regenerative medicine. These properties include: the ease of accessibility for isolation from the patients or bone marrow banks; the high expansion potential [46,47]; and, the presumptive plasticity, that is, being able to differentiate *in vitro*, not only into mesenchymal, but also non-mesenchymal lineages, including myoblasts [48], cardiomyocytes [49,50], hepatocyte-like cells [51], neuronal and neuroglial cells [52,53] and endothelial cells [54]. Moreover, when transplanted systemically, MSCs are able to migrate and home to the specific site of injury [55,56] and exert anti-inflammatory and immunosuppressive effects thus allowing a potential for their autologous and allogenic use [57,58,59]. Moreover, the absence of ethical issues concerning their tissue source and the lack of tumorigenicity represent additional advantages for their use in clinical applications [60,61].

### 2.2. Contribution of BM-MSCs to Muscle Repair/Regeneration

A large body of experimental evidence has shown that transplantation of BM-MSCs in animal models of muscle injury and disease has great therapeutic potential [6,62]. Indeed, the systemic or local administration of BM-MSCs into skeletal muscles subjected to traumatic injuries such as laceration [63], crush [64,65,66] or cardiotoxin injection [67,68], have been demonstrated to contribute to myofiber formation and to the functional recovery of the muscle tissue. A considerable augment in the capillary density and collateral perfusion, associated with a reduction of myofiber atrophy and disarray has also been observed in ischemic skeletal muscles transplanted with BM-MSCs [69,70,71]. Moreover, some studies reported that the injection of BM-MSCs into dystrophic muscles is capable to restore dystrophin expression [67,72,73,74], attenuate the oxidative stress [75], and improve the motor function [74]. Positive outcomes have been also obtained when BM-MSCs were utilized for treating post-myocardial infarction heart failure in both small (mouse and rat) and large animal (swine and dog) models. The administration of BM-MSCs (either by intravascular, intramyocardial or transendocardial injection), have in fact provided comprehensive functional benefits which include: attenuation of left ventricular negative remodelling with a reduction of the infarct size, increase of vascular density and myocardial perfusion [76,77,78,79,80,81,82,83,84], preservation of residual myocardium, and improvement of the contractile [76,77,78,81,85,86,87,88] and electrical properties [89,90]. However, despite these positive findings, there are controversies on the actual ability of BM-MSC to regenerate contracting myocardial tissue. Moreover, their retention and engraftment after transplantation into the diseased muscle still remain major limitations [76,77,91].

A number of clinical trials using autologous and allogenic BM-MSCs have been performed to improve the cardiac function in patients with acute myocardial infarction, or affected by chronic ischemic cardiomyopathy (Table 1). These studies have been proven to be generally safe and feasible without notable side effects; moreover, patients receiving BM-MSC therapy have experienced clinical benefits mainly in terms of scar reduction, a decrease in arrhythmias, an attenuation of ventricular contraction dysfunctions and an increase in left ventricular ejection fraction [92,93,94,95,96,97,98,99].

However, despite the encouraging outcomes, a deeper understanding of BM-MSC biology is required in order to validate their effective therapeutic benefits for muscle tissue repair/regeneration and elucidate the potential risks of their use in clinical applications.

### 2.3. Trophic Actions

There is a general consensus that the beneficial effects of BM-MSCs observed in animal models of human muscle disease, including acute and chronic myocardial infarction and dystrophy are mainly dependent on the trophic activity of the administered cells, rather than to their plasticity or stemness potential [100,101,102,103,104]. Many studies have shown, in fact, that MSCs do not differentiate into the cells of the injured organs and exert transient therapeutic effects in the absence of significant long-term engraftment (the injected cells are, in fact, rapidly lost after days from implantation) [105,106]. However, the implanted cells are metabolically and functionally active, capable of producing paracrine trophic factors with multiple effects in the host tissue microenvironment, including modulation of the endogenous repair mechanisms and prevention of injured cells from the stress response and apoptosis [106,107]. In this line, our group and others have shown that BM-MSCs produce, under normal culture conditions, a wide array of growth factors and cytokines [108,109] and can be activated to express and release higher levels of therapeutic factors in response to stress or inflammation signals [110,111]. While the administration of various single growth factors have demonstrated beneficial results in skeletal [112,113,114,115] and cardiac regeneration [116,117,118,119], the unique value of MSC-therapy resides in the possibility to obtain local, constant and biologically effective levels of different functionally synergistic trophic factors in the contest of the regenerating tissue and to achieve more sustained therapeutic effects. The best studied paracrine factors produced by BM-MSCs are those involved in the regulation of the innate immunity; accumulating evidence during the last years have, in fact, demonstrated that BM-MSCs have distinctive immunomodulatory and anti-inflammatory properties [58].

**Table 1:** Clinical trials using BM-MSC therapy for cardiac repair/regeneration.

Trial Name	ClinicalTrials.govIdentifier	Sponsor/ Collaborator	Location	Disease	Source	Route of Delivery	Patients	Status
Prospective randomized study of mesenchymal stem cell therapy in patients undergoing cardiac surgery (PROMETHEUS)	NCT00587990	National Heart, Lung, and Blood Institute (NHLBI)	USA	Chronic ischemic left ventricular disfunction	Autologous	Intramyocardial injection	45	Completed
		Johns Hopkins University Specialized Center for Cell Based Therapy						
Stem cell injection to treat heart damage during open heart surgery	NCT01557543	National Heart, Lung, and Blood Institute (NHLBI)	USA	Heart disease	Autologous	Intramyocardial injection	24	Recruiting
				Ischemic heart disease				
				Coronary artery disease				
Safety and efficacy of intracoronary adult human mesenchymal stem cells after acute myocardial infarction	NCT01392105	Yonsei University	Republic of Korea	Acute myocardial infarction	Autologous	Intracoronary	80	Completed
		FCB						
		Pharmicell Co Ltd.		(AMI)		injection		
Stem cell therapy for vasculogenesis in patients with severe myocardial ischemia	NCT00260338	Righospitalet, Copenhagen Denmark	Denmark	Myocardial ischemia	Autologous	Intramyocardial injection	31	Completed
		Jens Kastrup		Coronary heart disease				
Autologous mesenchymal stromal cell therapy in heart failure	NCT00644410	Righospitalet, Copenhagen DenmarkJens Kastrup	Denmark	Heart failure	Autologous	Intramyocardial injection	60	Recruiting
Prochymal® (human adult stem cells) intravenous infusion following acute myocardial infarction (AMI)	NCT00877903	Osiris Therapeutics	USA	Acute myocardial infarction	Allogenic	Intravenous injection	220	Active, not recruiting
			Canada	(AMI)				
Safety Study of AMI MultiStem® to treat Heart attacks	NCT00677222	Athersys, Inc	USA	Acute myocardial infarction	Allogenic	Via catheter into peri-vascular space injection	25	Completed
		PPD						
		Angiotech Pharmaceuticals		(AMI)				
A phase II dose-escalation study to assess the feasibility and safety of transendocardial delivery of three different doses of allogeneic mesenchymal precursor cells (MPCs) in subjects with heart failure	NCT00721045	Angioblast Systems, U.S.	USA	Heart failure	Allogenic	Trans-endocardial injection	60	Unknown (*last verified June 2010: active, not recruiting*)

This notion is remarkable, but not surprising; wide evidence, in fact, suggests that communication of BM-MSCs and cells of the immune system starts at the level of the bone marrow niche, where it play a crucial role in maintaining and preserving the undifferentiated state of the hematopoietic stem cells [41]. Recent *in vivo* and *in vitro* observations have demonstrated that the crosstalk between BM-MSCs and the innate immunity results from a combination of direct cell–cell contact and soluble factor-mediated mechanisms, including the release of molecules and bioactive metabolites with immunomodulatory action, such as interleukin (IL)-10, transforming growth factor (TGF)-β, galectin-1, galectin-3, leukemia inhibitory factor (LIF), nitric oxide and prostaglandin E2 (PGE2) [58]. A clear example of these interactions is the documented ability of BM-MSCs to mediate the transition of classically activated M1 macrophages into anti-inflammatory M2 macrophages, which participate in tissue healing, promoting the resolution of inflammation and the clearance of apoptotic cells [120,121]. The crosstalk between BM-MSCs and macrophages is further highlighted by the findings showing that BM-MSCs can prevent the release of tumor necrosis factor (TNF)-α and other inflammatory chemokines from activated macrophages, through the secretion of IL-1 receptor antagonist (IL-1RA) [122], and stimulate monocytes to release IL-1β, thus enhancing MSC-mediated secretion of TGF-β and the subsequent T lymphocyte suppression [123]. Beyond the inhibition of macrophage function, BM-MSCs also sustain the survival and the suppressive phenotypes of T regulatory lymphocytes, interfere with differentiation of dendritic cells and B lymphocytes, and inhibit proliferation and functionality of natural killer (NK) cells [124]. All these findings, together with the observation that BM-MSCs are immunoprivileged cells, owing to the low expression levels of HLA major histocompatibility complex (MHC) class I and co-stimulatory molecules, have raised the clinical interest in these cells, exploiting the possibility of a universal donor of BM-MSCs for therapeutic applications.

In addition to these effects, transplanted BM-MSCs may facilitate other complementary aspects of tissue repair, which, however, are considered prerequisites for tissue reconstitution and functional improvement and include augmentation of cell survival (limited apoptosis), promotion of angiogenesis and vasculogenesis, and inhibition of scarring (fibrosis). They are mediated in large part by a number of MSC-secreted factors which are capable of: (i) activating pro-survival pathways in the transplanted as well as resident viable cells ((IL-6 and IL-10, stromal cell-derived factor 1(SDF-1)) [125,126,127,128]; (ii) stimulating blood vessel growth (VEGF, fibroblast growth factor (FGF), IL-1, matrix metalloproteinases (MMPs), platelet derived growth factor (PDGF), TGF-β, angiopoetin) [129,130]; (iii) promoting favourable extracellular matrix remodeling (IL-10) and altering the passive characteristics of the scar [131]; and, (iv) inhibiting fibroblast–myofibroblast transition and the following collagen synthesis and deposition (hepatocyte growth factor –HGF, adrenomedullin) [132,133,134].

**Figure 1:**
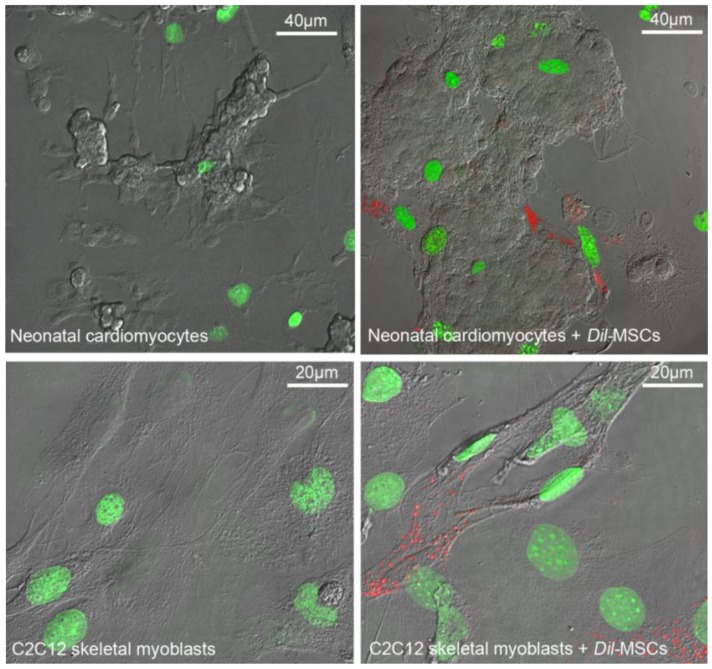
Bone marrow-derived mesenchymal stromal cells (BM-MSCs) stimulate proliferation of muscle progenitor cells. Representative superimposed differential interference contrast (DIC) and confocal fluorescence images showing the pyrimidine analogue EdU (5-ethynyl-2'-deoxyuridine) staining (green) in the nuclei of proliferating murine C2C12 skeletal myoblasts or murine neonatal cardiomyocytes in single and co-culture for 24 h with mouse Dil-labeled BM-MSCs (red). Note the higher number of proliferating cells in the co-culture systems.

Interestingly, it has been recently shown that BM-MSCs might affect cardiac or skeletal muscle repair via cytokine-induced enhancement of the host tissue (endogenous) stem cell function [135,136,137]. In this context, we have demonstrated in co-culture systems that BM-MSCs enhance neonatal cardiac as well as C2C12 skeletal myoblast proliferation (Figure 1) through a combination of juxtacrine and paracrine mechanisms that involve the activation of Notch-1 signaling [108,109]. We have also shown that these effects were mainly mediated by the release of VEGF by BM-MSCs [109]. Consistent with these findings, *in vivo* experiments have shown that injection of BM-MSCs promote the activation of muscle satellite cells and the formation of new myofibers in the injected skeletal muscles [75]. Moreover, the engrafted BM-MSCs can directly participate in the recruitment and differentiation of the endogenous cardiac progenitor cells in the diseased myocardium, via the paracrine release of SDF-1α, a potent chemoattractant for stem cells [127,138].

### 2.4. Strategies to Advance the Therapeutic Properties of BM-MSCs

Much emphasis is currently given to the identification of new strategies to optimize the cell fate after *in vivo* BM*-*MSC administration. These techniques target a wide array of biological functions, including the cell homing, survival, proliferation and paracrine secretion. Studies have shown that preconditioning by exposure to reduced levels of oxygen, incubation with nitric oxide, hydrogen peroxide or diazoxide, and treatment with pharmacological drugs, including phosphodiesterase inhibitors, angiotensin II receptor blocker and neuropeptide Y, may greatly enhance the therapeutic promise of BM-MSCs [44]. Moreover, it has been recently shown by our group that the treatment with platelet-derived rich plasma (PRP) [139], as well as irradiation with low level lasers [140], represent promising preconditioning approaches for stimulating BM-MSC proliferation, encompassing senescence during cell expansion and influencing stemness gene expression. Of interest, the genetic manipulation of MSC to overexpress cytokines and growth factors, such as HGF, VEGF and SDF-1, have also been proposed to improve neo-angiogenesis and the endogenous mechanisms of tissue repair/regeneration [44,141,142]. Finally, bioengineered three-dimensional matrices have been shown to represent appropriate devices to preserve the survival of the engrafted MSCs and assist their migration within the damaged tissues [139,143,144]. 

## 3. Conclusions and Future Perspectives

**Scheme 1:**
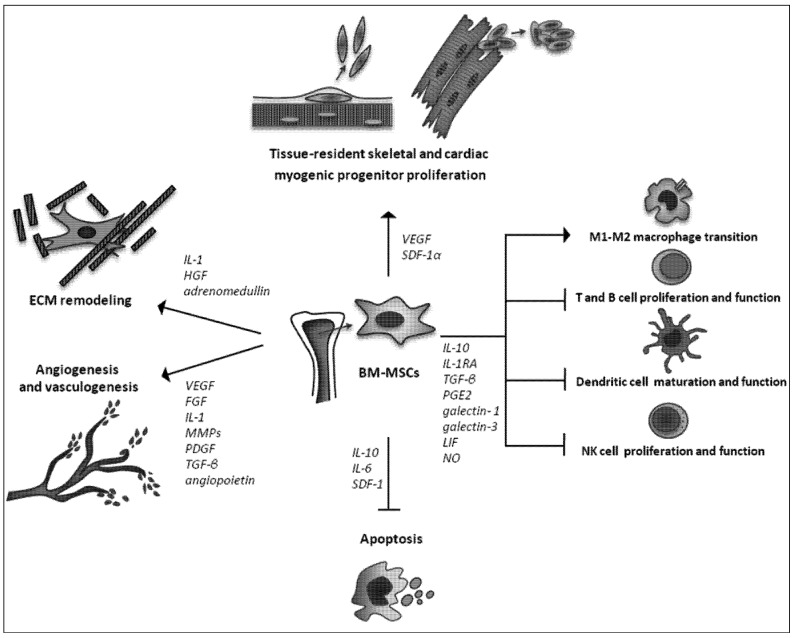
Therapeutic paracrine effects of bone marrow derived mesenchymal stromal cells (BM-MSCs) for skeletal and cardiac muscle repair/regeneration. *Abbreviations*: ECM: Extracellular matrix; FGF: Fibroblast growth factor; HGF: Hepatocyte growth factor; IL-1: Interleukin 1; IL 6: Interleukin-6; IL-10: Interleukin 10; MMPs: Matrix metalloproteinases; PDGF: Platelet derived growth factor; TGF-β: transforming growth factor-β; VEGF: Vascular endothelial growth factor; SDF-1: stromal derived factor-1; IL-1RA: interleukin 1 receptor antagonist; PGE2: prostaglandin E2; LIF: leukemia inhibitory factor; NO: Nitric oxide.

BM-MSCs are considered among the best candidates for cell-based therapy for skeletal and cardiac muscle repair/regeneration. Their beneficial effects are mainly related to the release of a wide range of trophic factors with multiple effects in the host tissue (Scheme 1). Of particular interest are the findings showing that BM-MSCs stimulate proliferation and differentiation of the resident muscle progenitors, providing novel concepts for considering these cells as instructing and supporting elements capable of modulating the endogenous tissue repair mechanisms. *In vitro* priming of BM-MSCs by a wide variety of techniques may be used to complement the biological and biochemical properties of these cells and allow the expansion of their therapeutic potential. However, at present, the clinical application of BM-MSCs is considered with caution and long-term studies are still required in order to elucidate the side effects and validate the safety of these cells for tissue regeneration.
